# “Ashamed, Silent and Stuck in a System”—Applying a Structural Violence Lens to Midwives’ Stories on Social Disadvantage in Pregnancy

**DOI:** 10.3390/ijerph17249355

**Published:** 2020-12-14

**Authors:** Eva Neely, Briony Raven, Lesley Dixon, Carol Bartle, Carmen Timu-Parata

**Affiliations:** 1 School of Health, Te Herenga Waka—Victoria University of Wellington, Wellington 6140, New Zealand; 2 Maternity Equity Action, Haumoana 4102, New Zealand; maternityequityaction@outlook.co.nz; 3 New Zealand College of Midwives, Christchurch 8014, New Zealand; practice@nzcom.org.nz (L.D.); policyanalyst@nzcom.org.nz (C.B.); 4 Ngati Kahungunu, Department of Public Health, Otago University, Wellington 6242, New Zealand; carmen@nzba.co.nz

**Keywords:** maternal health, structural violence, equity, social disadvantage, pregnancy, midwifery

## Abstract

Historical and enduring maternal health inequities and injustices continue to grow in Aotearoa New Zealand, despite attempts to address the problem. Pregnancy increases vulnerability to poverty through a variety of mechanisms. This project qualitatively analysed an open survey response from midwives about their experiences of providing maternity care to women living with social disadvantage. We used a structural violence lens to examine the effects of social disadvantage on pregnant women. The analysis of midwives’ narratives exposed three mechanisms by which women were exposed to structural violence, these included structural disempowerment, inequitable risk and the neoliberal system. Women were structurally disempowered through reduced access to agency, lack of opportunities and inadequate meeting of basic human needs. Disadvantage exacerbated risks inequitably by increasing barriers to care, exacerbating the impact of adverse life circumstances and causing chronic stress. Lastly, the neoliberal system emphasised individual responsibility that perpetuated inequities. Despite the stated aim of equitable access to health care for all in policy documents, the current system and social structure continues to perpetuate systemic disadvantage.

## 1. Introduction

Historical and enduring maternal health inequities and injustice continue to grow in Aotearoa New Zealand, despite attempts to address the problem [1,2,3]. The Director General of Health stated that “in Aotearoa New Zealand, people have differences in health that are not only avoidable but unfair and unjust. Equity recognises different people with different levels of advantage require different approaches and resources to get equitable health outcomes” [4].

Aotearoa has a maternity system that provides continuity of midwifery care for all eligible women, which aims to provide seamless, high quality and equitable care. This model of care increases maternal satisfaction and reduces preterm birth whilst supporting safe outcomes [5,6]. Further, evidence shows that needs-led caseload midwifery care built on mutual trust is particularly beneficial for socially disadvantaged women [7,8,9]. However, women living in socioeconomic deprivation continue to face barriers to care and difficulties navigating the maternity system [1], with access to midwifery care described as “daunting” or the service considered a “maze” by some women [10]. Survey data from the Growing Up in New Zealand longitudinal study found that women who were non-European, younger, in their first pregnancy and living with greater socioeconomic deprivation registered later for their antenatal care [11]. Further, for many Māori, the current public health system is experienced as hostile due to barriers within the organisational structures such as staff interactions and practical considerations [12]. Despite clear evidence of inequities and increased care needs for Māori wāhine (women), addressing and improving service access and delivery requires adequate resourcing, which has not been prioritised, allowing inequities to persist and women’s rights to be eroded [13,14].

Pregnancy increases vulnerability to poverty [8,15]. Living in poverty often means living without and this can include living without essential health care due to costs, barriers to access or gaps in service provision. In 2017, the Report on Maternity identified that 22.3% of women who gave birth lived in the most deprived quintiles, with Māori (48.5%), Pacific (59.8%) and Indian (31.9%) ethnicities over-represented [16]. These same groups, and specifically women in lower socioeconomic groups, are also over-represented in mortality statistics and have higher maternal mortality and perinatal mortality (stillbirth and neonatal death) (Perinatal and Maternal Mortality Review Committee, 2019). Midwives working in continuity of care with women are well positioned to recognise and address issues of poverty affecting women and whānau (loosely translated to kin and nonkin family).

Key barriers to equitable maternity care in New Zealand include the neoliberal political climate, physical access to services, equity of choice in maternity care provider as well as access to secondary care when needed, acceptability of the service for socially disadvantaged women, colonial structures of loss, displacement and marginalisation inhibiting self-determination; and ethnic, racial and cultural discrimination [1]. These systemic and structural issues have a greater impact on pregnant women living with social disadvantage, exacerbate inequities and contribute to further intergenerational transmission of trauma and disadvantage.

International studies suggest that even when there are efforts to improve marginalised women’s health, actions can often generate feelings of stigma and judgement towards individualized failure and mask the system level barriers that exist in women’s lives [17]. Closer examination of socially disadvantaged women’s everyday realities reveals complexity, which includes the impact of material deprivation, precarious social support, lack of control and volatile partner relations, increasing the strain on mental and physical health [8,15]. Scaling up from the everyday to the systemic, Solnes Miltenburg et al. [18] examine how gender inequality legitimises and perpetuates abuse towards women in maternity care and therewith highlights the harm institutionalised practices and policies cause for vulnerable, pregnant women. Unequal access to the social determinants of health (e.g., housing, unemployment) further increase the risk of interpersonal violence for women [19], with particular likelihood of causing not only physical and mental health harm to women but also their baby and impacting on a woman’s ability to secure the custody for her own baby [20,21].

### 1.1. Aotearoa Maternity Model of Care

Maternity care in Aotearoa New Zealand adheres to four key principles. Firstly, supporting women and whānau to have a fulfilling childbirth outcome; secondly. recognition that pregnancy and childbirth are normal life events; thirdly, provision of continuity of care through a lead maternity carer (LMC) as primary care giver responsible for assessment and care planning with the woman and lastly, facilitation of additional care for women and babies who require it [22].

When a woman becomes pregnant she chooses a care provider to be her LMC (this can be a midwife, general practitioner or obstetrician) with the majority choosing a midwife [16,22]. The LMC provides care to the woman and her whānau in the woman’s community including postnatal visits to her home with additional care provided by obstetricians/physicians in the secondary/tertiary maternity hospital—for those with complex pregnancies or risk factors. The LMC provides continuity of midwifery care for pregnancy, labour and birth and for up to six weeks following the birth. This model of care has been shown to increase women’s satisfaction and results in fewer caesareans sections and instrumental birth, less use of epidural and pharmacological pain relief, shorter labour and more babies with higher Apgar scores [5,23,24,25].

Midwives in New Zealand can work as an LMC in the community with a caseload of women (contracted by the Ministry of Health to provide services (as per Section 88 Maternity Services) or they can work as hospital midwives, working primarily with women who have been admitted for hospital care during pregnancy, childbirth and postpartum. Midwifery in Aotearoa New Zealand is based on a partnership model of care which emphasises equality, empowerment, negotiation, shared responsibility and informed consent.

### 1.2. Theoretical Lens

Structural violence is an analytical tool used to examine and reveal the enduring and perpetuating effects of colonisation, neoliberal economies and global structures of oppression. In this sense, it examines how violence is built into structures, ideologies, institutions and histories and “manifests itself as inequality of power, resources, and life opportunities” [26] (p. 195). Its function is to reframe disadvantaged and disenfranchised populations and in health research is used to focus on the deep structural roots of health inequities. Galtung [27] draws on a health example to illustrate structural violence: “if a person died from tuberculosis in the eighteenth century, it would be hard to conceive of this as violence since it might have been quite unavoidable, but if he dies from it today, despite all the medical resources in the world, then violence is present according to our definition” (p. 168). Galtung [15] thus sees structural violence as social injustice as a social order that causes avoidable and unnecessary suffering.

Structural violence, in contrast to social determinants of health, helps us move from a passive towards an active doing of injustice through perpetuating health inequities [28]. It helps us see beyond the personal, direct violence caused by an individual towards the indirect, embedded and systemic violence often otherwise framed as natural suffering. Structural violence may also help reveal hidden assumptions of colonialism that have shaped current knowledges on the needs of indigenous populations [29] and revert the argument of biological determinism for the cause of indigenous deaths [30]. A structural violence lens can lead us away from framing perpetual deficiencies towards making space for new knowledges on the causes of “impoverished material conditions [that are] structured by politically oppressive regimes” [29]. Gamlin and Holmes [31] for instance show that a structural violence perspective has the ability to counter the common presumption that the “choices of indigenous women to avoid institutional delivery are irrational, cultural or due to a lack of education” (p. 1) and reveal that these are instead due to institutional and systemic forces that lead to preventable deaths.

In this study, we have used the concept of structural violence to examine how midwives have described the circumstances and maternity care of pregnant women living in socioeconomic disadvantage. The recategorisation from suffering to harm enables us to view women’s circumstances as a failure by the state and their risks as accountable to societal inaction and has been used to examine maternal inequities effectively [18,19,32]. Thus, by “foregrounding forms of violence that go unnoticed and unrecognized”, we aim to expand “the field of visibility and registers forms of invisible violence” [14] (p. 198). Our rationale for choosing structural violence to examine the impact of poverty on pregnant women is threefold. First, structural violence enables us to understand how institutionalised social structures remove agency from women in invisible and subtle ways. Second, it enables us to examine how women living with disadvantage are actively exposed to greater risk through systemic processes. Finally, it allows us to view the violence built into the neoliberal system of the state through repetition and routinisation that are integral to the social organisation.

## 2. Materials and Methods

This study drew on the final open-ended question of a survey distributed to midwifery members of the New Zealand College of Midwives in 2018. The online self-report survey topic was midwifery perspectives on the impact of maternal disadvantage on maternity care (results in press). Most survey items were quantitative with some open-ended questions, enabling midwives to illustrate their thoughts through stories. A final question asked the respondents to “share a story from a time in the last year where a woman you cared for was disadvantaged.” Responses to this question ranged from a few words to detailed narratives. This paper details an indepth exploration, using a structural violence framework to identify the themes provided in the response to this question. Text responses have a participant number identified from the excel data sheet. Spelling mistakes have been rectified but grammatical mistakes have been left to ensure that the intent of the response remains intact.

We received 436 survey responses of which 214 midwives shared a story (49.2%). Of the midwives who provided stories, 126 (58.8%) were community midwives, 29 (13.5%) were hospital midwives and 59 (27.5%) were in “other” roles.

Within the survey, maternal disadvantage was primarily characterised as transient housing, homelessness, heating and weatherproofing of homes, low income, food security and social issues. The open-ended question encouraged midwives to describe examples from practice and to explore wider notions of poverty.

### 2.1. Data Analysis

We drew on an inductive thematic analysis approach to survey data [33] and were theoretically informed by structural violence [26] in our theming and framing of the issues arising from the data. In the first instance, we descriptively coded the data. This reading of the data resulted in 91 codes, which we then examined through a structural violence lens including institutionalised social structures, exacerbated risks and neoliberal systems. All authors independently read through the text data responses. Through an inductive and iterative approach, we examined the issues midwives brought up as caused by structural violence. The final themes were agreed through discussion amongst the authors.

### 2.2. Ethical Considerations

The project was assessed by the Massey University Ethics committee and identified as low risk (Ethics identification number 4000020305).

## 3. Results

Using the structural violence lens, we identified three themes that illustrated the intersection of pregnancy and poverty: structural disempowerment, inequitable risks and neoliberal system. The core ideas of each theme can be found in Table 1.

### 3.1. Structrual Disempowerment

The first theme reflects the ways in which women experienced structural disempowerment in patterned and predictable ways that actively worked to create barriers. In the narratives, midwives described situations where women experienced reduced agency with little opportunity to improve their own situations.

*Poverty makes people ashamed, silent, and stuck in a system where risks are increased. It drains everyone’s energy and trigger as well frustration with a system so complicated to access, fragmented, or unable to provide continuity of care.*

(Participant 159)

Midwives observed that despite seeking agency and personal empowerment over their lives, women were met with disempowering structures produced through disadvantage, stigma and discrimination.

*Briefly cared for a woman whose baby was removed from her care due to uncontrolled epilepsy. This woman was living in poverty and had not had a seizure for a long period of time. This was versus another woman who had a baby at a similar time, not in poverty but controlled epilepsy and had a seizure in the early days following the birth of her baby. The woman without poverty kept her baby in her care and was supported to do so, the woman living in poverty wasn’t given such support or a choice about what happened to her baby.*

(Participant 45)

*It’s just everything. They don’t have transport or childcare, so they can’t make it to strictly timed [obstetric] appointments or to be assessed in a timely way. They can’t afford to feed themselves with nutritious food. We can treat their STIs but they’re recurrent because they’re partner won’t or can’t afford to get treated.*

(Participant 101)

Midwives described how women who were unable to attend appointments were often considered within the system to be nonattenders and/or noncompliant. In their stories, midwives often shared their perspectives on how disadvantaged women faced particular challenges by having reduced agency. Midwives indicated that sometimes women expressed that they saw pregnancy as an opportunity to improve their own health, for the sake of the baby. The continuity of care model meant that midwives were often contextually knowledgeable about the intricacies of women’s lives and witnessed how women struggled to achieve agency to improve their lives due to their lack of resources.

*A woman had limited funds and a strict budget which meant she missed clinic appointments with the diabetes service due to costs involved i.e., gas, childcare, parking etc. She was written off as a non-attender and a report of concern was placed on her.*

(Participant 23)

*Most women I care for, know that maternity care is free in NZ. They do access care. The inequity comes in the form of judgement from some hospital providers who expect these women to be highly functioning when often it is a struggle for women to face a stranger and let them know what’s happening.*

(Participant 130)

The midwives’ stories revealed that sometimes decisions were made through prejudice and stigma rather than objective assessment. These decisions had profound impacts on the woman resulting in a reduction in services and support, which had potential to increase the risk of family breakdown or even uplifts of babies to state care.

*I am currently looking after a woman in pregnancy, who has previously had children uplifted. She lives with violence and poverty. She struggles also with alcohol. Social services are not willing to provide additional support as they say they have done before and the change was not sustained. The plan is to uplift this baby too. No agencies seem to want to help her, or to help me help her.*

(Participant 67)

Women were further structurally disempowered through their positions within families. Quite often it appeared that women had reduced access to resources that existed in the family, which could come at a cost for her own health and access to pregnancy related needs.

*One couple in their mid-twenties with little money and no car, LMC (midwife) had to pick them up from their home and bring them to the Birthing unit in labour. He had spent the money they were saving for a car at the pub. She had no sanitary items and few clothes for the baby. LMC was very supportive but it was extra work and worry for her.*

(Participant 124)

In many instances, women did not have basic human needs met, such as food, shelter and safety. Not only do these needs prohibit women from meeting their psychological and self-fulfilment needs but these are also fundamental human rights that are required for a pregnant woman to be safe from violence and to thrive in her pregnancy.

*Woman living in remote rural location. Being supported by women’s refuge due to history of domestic violence in previous relationships resulting in loss of care of previous children. Partner heavily involved in gangs. Women has limited access to money. Reliant on partner for transport but partner needing car for work so unable to easily get to appointments (midwife, bloods, scan). She (was) often dropped at his mothers where I would visit her. No safe place to talk without being overheard by partner/his mother.*

(Participant 199)

The midwifery narratives identified that many women were not able to access basics (e.g., housing, transport and phones) to achieve wellbeing for themselves and their babies. These systemic barriers appeared to further exacerbate disempowering life circumstances for many women.

### 3.2. Inequitable Risk

The second way in which structural violence was executed was by exacerbating risks for women through intersectional disadvantages. Across the stories it was evident that women experienced additional layers of disadvantage when becoming pregnant, which ultimately put them at greater risk. Pregnancy thus confounded existing, and created additional risks, for disadvantaged women disproportionately.

*A woman I cared for on the ward presented to birthing [suite], unbooked and in labour. She had not been able to seek midwifery care as her phone had no credit or she lost [it], she was transient and currently staying with her mother who was not allowed extra tenants in her house. She had not had a scan since a dating scan and nil current bloods. She then required follow up with the social worker while in hospital and will then receive social support postnatally and be supported with this by a midwife. However, if she had been able to access and engage with care antenatally she may have been able to have had those supports in place prior to birth.*

(Participant 24)

It was apparent that the women midwives provided care for in our study had increased stress levels and mental health concerns, family violence and abuse, exposure to addictions and drugs, with poverty increasing the medical risks they were exposed to throughout pregnancy via multiple pathways. Despite their heightened vulnerability in pregnancy, women did not appear to receive additional support to counter the inequities.

*Women in poverty need priority care in our system. Their diets are poor and they are more likely to use nicotine or other substances, leading to poor outcomes for their own and their baby’s health. They avoid medical and dental services due to the costs so they are constantly disadvantaged with their health. The repercussions include mental health issues and the mental health services seem to be only able to prioritize the most unwell, so often mental health issues are not properly addressed either.*

(Participant 229)

Although New Zealand’s maternity system is publicly funded there were evidently a range of barriers for disadvantaged women when accessing maternity care. These included surcharges for ultrasound scans and prescriptions. Missed screening can increase medical risks in pregnancy. These barriers can generate a range of pathways of risk that directly and indirectly mean women have a higher risk for medical complications, independent of pre-existing conditions, ethnicity or age. This is one example of how poverty itself can be seen as a risk factor for complications for mother and baby. Furthermore, these responses demonstrate how poverty creates chronic stress in women’s lives, exposing them to multiple pathological impacts on them and their babies.

*Poverty is very real. Women are trying to do their best in such poor conditions and they are constantly having to fight the systems that are meant to be there serving them, advocating for them, and supporting them. Judgements are made about women and their previous poor decision making... they are trying to do their best for their babies.*

(Participant 230)

Midwives described that women often lived with chronic stress, which was exacerbated when dealing with the physiological and psychological effects of pregnancy, increasing the toll on women’s mental health. Women had additional worries such as changing housing, insecure or unsafe employment, physical safety or lack of access to childcare to attend appointments, which accumulated in stress across prolonged periods.

*A woman with DNA [did not attend] history and mental health problems was asking for some hours of childcare so she could take time for herself and didn’t have to “lose it” with the baby- nothing at all was available and she ended up having more stress as the friend she was leaving her baby with when she was overwhelmed was not a good choice.*

(Participant 14)

*That same day I saw a woman who had to shift house in the first week of having a baby into emergency accommodation which was a one room complex with her and her three children and partner and sister!*

(Participant 42)

From the stories it was evident that many women had previous mental health issues, while for others pregnancy triggered poor mental health. Regardless of the origin, it appeared to be a common narrative that neither women nor midwives could access the appropriate mental health support required. The same was apparent with addiction services.

*Mental health issues from rape trauma. Getting support in the community was very difficult to access. She was treated without compassion or kindness based on how she presented herself. After birth baby was removed and she was discharged an hour later. No follow up support from mental health staff.*

(Participant 127)

*I have so many stories it is hard to choose. The most common issues are mental health, depression, anxiety, PTSD (not uncommonly from birth experiences) psychosis, bi-polar. There is so little help or places I can get help for these woman.*

(Participant 88)

Family violence exponentially exacerbated risks in pregnancies and intensified interpersonal and structural violence. However, it was apparent that under midwifery care, the woman was more likely to disclose and receive help because they developed a relationship with their midwives who were able to act as advocates.

*1st baby, approximately 24 wks. No other antenatal care at this stage. Lots of DNA’s [did not attend] to get to this appointment. Hence why a home visit was offered. Outside my usual practice. The woman was difficult to get eye contact with. Behaving stressed and distracted. Looking at the clock a lot. Noted to have some bruising on her neck. Within 5 min of starting the appointment I asked if she was ok as didn’t seem ok. And what the marks on her neck were. She immediately starting crying …she was worried about me coming today. But also relieved as knew she needed help. She said he was coming home soon and didn’t feel safe… she come in my car and we drive away. We drove to somewhere safer... immediate contact with woman’s refuge…She was desperate for help. Before she left I did wrap around service referrals.*

(Participant 68)

Midwives sometimes acted as a facilitator to help women get out of unsafe situations. Issues like pregnancy loss often added an additional layer of strain on families who were struggling already. In one instance the midwife described grief due to baby loss, that increased the stress for a woman who was not granted the privilege of recovery time.

*Last month, a woman we’re caring for lost her baby at 28 weeks. It feels important to acknowledge that all of the compounding risk factors this beautiful woman had for such a tragedy, can all be linked to the health disparities associated with Te Tiriti o Waitangi (The founding document of New Zealand which states the legal relationship between Māori and Pākehā). Just prior to conceiving this baby she was admitted to ED as a result of domestic violence. Furthermore, she’s living in emergency accommodation, in a motel with her husband and their 18-month old daughter. He is a laborer and she looks after their baby. Amongst their grief, they were still expected to continue to prove their income, with the system requiring them to show they’ve tried to actively but unsuccessfully source at least three different private rentals before they truly qualify for state housing.*

(Participant 101)

In this instance, not having privilege (such as housing security, physical safety) meant that there was a need to deprioritise grief and jump through systemic “hoops” to prove the need for support. Additional strains on under-resourced women causes further disadvantage and may exacerbate risks leading to post-traumatic stress disorder. Recurrence of such issues feeds into an intergenerational cycle of distress and trauma that accumulatively exacerbates risk further.

### 3.3. Neoliberal System

The third theme is concerned with how existing systems are set up to rely on individual action, advocacy and persistence to function. It was evident from the midwives’ narratives that the current system could perpetuate disadvantage for women. The system appeared to rely on the concepts of autonomy and personal responsibility; however, for women experiencing disadvantage, the system was prohibitive to achieving this agency and personal advocacy.

*Its not inequity in the maternity system, its inequity in day to day living. This then impacts the woman’s ability to attend appointments, have the ability to pay for scans, scripts and needs for the newborn and mother; and also transport to attend appointments… This what needs [to be] addressed not the maternity system.*

(Participant 115)

Many women did not attend (DNA) their appointments with midwives, sonographers or doctors. Midwives’ stories revealed that missing appointments was however more frequently due to a lack of resources to attend the appointment. This a could be described as a reframing of DNA to WNA (was not able)—due to a system that is reliant on adequate resources to function.

*She had to travel to appointments which she could not afford, the second appointment which was to see the psychiatrist, was cancelled due to the Doctor being sick. However she was not notified and had travelled about 40 km to this appointment using petrol she could not afford. When it was rescheduled another week later, she could not afford to go.*

(Participant 3)

The greater travel distances were often due to a lack of specialist services in rural centres and the need to travel further. New Zealand is geographically diverse with many rural and remote rural populations. This further reduces access to maternity services for women and their families. Midwives described the issues as:

*Equity does not exist for women from rural areas at all. Marked as non-attenders when appointments are made that they cannot get to for reasons beyond their control.*

(Participant 58)

*Often rural women who need obstetric review cannot afford to get to appointment at hospital so will DNA. Would be helpful to have obstetric clinic in their community.*

(Participant 144)

In these circumstances, midwives explained that they would often provide antenatal home visits and sometimes provided transport for external appointments (this is not funded). Access to support services were also described as inequitable for some women.

*I have a predominately high case load of young Māori; who face enormous inequities within the system. They hit the ‘risk’ categories that require referrals to services not specific too them, so they do not attend as we did not inspire… I have seen these woman get less support than the average aged Pākehā [New Zealanders primarily of European descent] women.*

(Participant 82)

In these situations, the system (including systematic racism) would appear to have failed to meet the needs of women. Midwives’ narratives describe a system consisting of multiple barriers including access to health services, barriers to funding and barriers to treatment. Socially disadvantaged women were exposed to a range of risk factors because of a system that is designed to serve those with the resources and skills to navigate themselves through.

*A woman with a urinary tract infection could not pay for her prescription. She developed pyelonephritis. Went into preterm labour at 33 weeks. Baby required admission to the neonatal unit.*

(Participant 35)

Inequity was often experienced prior to pregnancy and was compounded through the lack of available funding, services and supports within the system.

*No resources... too young to receive benefit, too young to drive…, unable to get to hospital for appointments, no phone for us to contact her to arrange visit... when we went (she) had not eaten for 4 days as no money for food.... social services couldn’t help too young refer to different agency etc. we felt agencies waiting to pounce instead of wrap around support services. we would take food and resources, collected clothes from donations to maternity unit, gave her pēpi pod [bassinet], she breastfed beautifully... we later learned Oranga Tamariki [Ministry for Children] uplifted her baby.*

(Participant 173)

There was a lack of resourcing in the system to ensure women’s safety and which can result in more family breakdowns including uplifts of children.

*This māma was terrified and tried so hard to “tick the boxes” to have been allowed to keep her babies. There was no support but her social work notes advised ongoing observation and consideration of uplifting the babies if the family were found to be dangerous: realistic alternatives for this woman did not exist in her world. She had nowhere else to go.*

(Participant 223)

The midwives explained that they provided care to women who lived in unclean and damp housing, frequently faced threats to housing security, and were at times also homeless. It is important to note that the women described by the midwives were receiving midwifery care, albeit under constrained circumstances, and this was enabled by a midwifery-led continuity model of care. Midwives worked hard to support disadvantaged women despite lack of funding and resources.

*The interventions we make at this juncture in women’s healthcare is the ambulance at the bottom of the cliff. GP [General Practitioner] services are lacking and many can’t afford to access them. Housing is poor and social deprivation, drug & alcohol abuse, smoking, family violence and child abuse are rife. No matter how much intervention we make during the maternity phase of life it is frustrating that sometimes we can really make very little difference. The problem is much bigger on a societal level than maternity care can deal with alone.*

(Participant 231)

The system perpetuated structural violence further through prioritising individual and biomedical health needs. Midwives often attempted to access appropriate resources for women; however, the system was not geared up to service women’s holistic and whānau/family wellbeing needs.

*A woman with mental health issues and using P [Methamphetamine] with an extremely low BMI (15) with 4 children (only two of them in her care) and pregnant for a 5th time was denied permanent contraception through her DHB [District Health Board] despite myself and the GP writing letters after the birth of her 4th baby to say that she really could not cope with any more pregnancies. The 5th pregnancy she was sent from pillar to post before she could find someone who would refer her for termination of her pregnancy.*

(Participant 35)

Lastly, it was evident through the increased risk women faced through stigma and discrimination how the system further perpetuated women’s circumstances through institutionalised racism.

*Māori women experience more disadvantage as a population due to the ongoing results of colonisation. I recall one woman who was recommended to stay in hospital overnight for assessment of abdominal pain due to a concern over the possibility of placental abruption. She felt insecure staying on her own at the hospital and said she would stay if a whānau [family] member could stay with her. I asked for this to happen in order to facilitate appropriate clinical and culturally safe care. The associate charge midwife said this would not be possible as the woman would be in a shared room. I spoke to the registrar who said ‘this is the care we are offering. If she doesn’t want it… she can self-discharge against medical advice’. I have since seen partners/family members being allowed to stay with (Pākehā) women in shared rooms provided the neighbouring woman consented to this. The woman in question discharged home to be with her whanau as the hospital would not facilitate her to stay with a whanau member. This put the woman at risk of an adverse outcome.*

(Participant 5)

## 4. Discussion

Using a structural violence lens facilitated improved comprehension of the dynamics of poverty and enabled us to view women’s circumstances as a failure by the state. Thus by “foregrounding forms of violence that go unnoticed and unrecognized” we aimed to expand “the field of visibility and register forms of invisible violence” [14] (p. 198). Using the structural violence lens framework on midwives’ narratives uncovered three interrelated themes, including structural disempowerment, inequitable risk and the neoliberal system. Similar to other structural violence research our analysis was able to indicate structural-level factors that exacerbate inequities and produce reproductive injustices. Similar to Milaney et al. [34], we could see patterns of gendered injustices, structural barriers to receiving support and disconnected services that exacerbate trauma. Adding longitudinal social network analysis might enable more nuanced perspectives on root origins of inequities and offer ideas for solutions [32] that tie structural violence to a broader theoretical framework and can offer a stronger mandate for action [28].

In Aotearoa New Zealand, the present-day maternal health inequities are an embodiment of accumulative displacement, adversity and neglect created through policies and colonisation across the last centuries. The colonisation of New Zealand in the 1800s resulted in a dominant Pākehā model of governance [2,12,35] and knowledge generation [29]. This has led to a model of health that is focused on individual responsibility, which is at odds with indigenous understandings of holistic health situated within a community [31,36]. At its core, New Zealand’s unique midwifery-led continuity of care model incorporates a holistic philosophy encompassing the wellbeing of the woman and whānau/family. The midwives’ narratives demonstrated how this holistic approach as well as the women’s needs often clashed with the neoliberal philosophy dominant within the health system resulting in women’s inability to access required medical care and social support. It is evident that colonization and neoliberal reforms from the 1990s continue to influence maternity care and women’s experiences of that care in contemporary New Zealand.

Structural violence exists in interdependence with interpersonal violence, with the interpersonal encounters of physical violence often being perpetuated through invisible structural violence worsening women’s situations [19]. However, by turning a more direct gaze towards the invisible we were able to examine differential access to power and resources as a form of violence and shift the “category of violence away from surface phenomena toward a broad set of social relations” [14] (p. 195). We can therewith go beyond the individualist empowerment narrative and see how structural determinants, invisible mostly to the public eye, constrain women in their agency. We can see from the midwives’ narratives how structural violence manifests as unequal access to systems of support such as welfare, housing or health care. We know that this results in a vicious cycle since women are more likely to be dependent on others and trapped in poverty with limited opportunities to escape [19]. The intergenerational transmission of disadvantage exacerbates the structural violence in which women are set up to fail [18,19,37]. The midwives often identified nuances of women’s adverse early life experiences in their stories, exposing how these experiences appeared to have carried on into women’s adult lives. This is supported by a large body of evidence on the ongoing effects of adverse early life experiences and the urgent need to add resources to break the cycle [38,39,40].

Pregnant women living in deprivation were exposed to greater physical and mental health risks because of societal failure to address women’s needs equitably. This is concerning given the evidence on the effects of this risk exposure over decades [41]. The first risk exposure pathway operates via barriers to service provision, which means that socially disadvantaged women receive less care and preventable complications are more common [1,12]. Secondly, the neurobiological effects of living with poverty increases the risk of chronic stress, which perpetuates violence towards women in the form of mental illness and addictions [42]. Our findings resonate with the broader literature on chronic stress effects and transmission to offspring. For instance, in a study with drug dependent mothers, Sánchez-Sauco et al. [32] observed how feelings of loneliness and hopelessness are structurally fostered and reproduced for socially disadvantaged mothers. Further, these risks may be exacerbated in Aotearoa New Zealand by factors such as physical access, affordability, cultural acceptability and pre-existing conditions that have shown to be predictors of maternal health inequity [1].

The midwifery-led continuity model benefits women living in social disadvantage because (as the narratives demonstrate) the midwives have invested extra time, resources and emotional support [7,8,43,44]. This can be seen in the narratives from the study participants, despite inadequate funding in the current Aotearoa New Zealand model. While there are key issues that need to be addressed such as workforce development and sustainability and more Māori and Pacific midwives to serve as midwives in their communities, it is evident that midwives’ relationships with women reveal great insight and understanding of women’s lives. In a Canadian population-level, retrospective cohort study of pregnant women in British Columbia (BC) by McRae et al. [43] antenatal midwifery care was linked to better outcomes for women of low socioeconomic position. The authors examined maternity care outcomes for 57,872 women under the care of general practitioners, obstetricians and midwives, and found lower odds of small for gestational age birth (SGA), preterm birth (PTB) or low birthweight infants (LBW) for women of lower socioeconomic positions who received antenatal midwifery versus physician-led care. It is difficult to estimate the impact that midwifery care has on the current outcomes for New Zealand, although it is apparent that women living in socioeconomic deprivation continue to have poorer outcomes [16,45]. Clearly, there is a limit to the impact the midwives can have without more comprehensive and systematic government and agency support.

The current health system is founded on expectations of individual responsibility, choice and autonomy but for socially disadvantaged women, pregnancy can be experienced as a source of disempowerment, exacerbated risks and reduced choice. Issues a well-resourced person may see as inconveniences can lead to reduced healthcare for disadvantaged women. Cancelled appointments, copayments or travel distances are less likely to be barriers for the well-resourced woman who can make a new appointment, advocate for their need or drive a distance for a particular service. In the midwives’ narratives, it was apparent that some women often did not have sufficient income to be able to pay the copayments for ultrasound scans or prescription charges. They also often had no transport or childcare meaning that they were unable to attend an appointment. As a result, and within the hospital system, they were often labelled, with no attempt to understand the individual woman’s context and little follow-up or support to ensure ongoing care. These findings align with existing research that impose neoliberal values on health system use and create barriers to health [1,35,46]. Scholars have exposed the damage such systems can have on indigenous populations in which these conflicting values systems lead to long-term mortality and trauma increases [31,47]. Gamlin and Hawkes [48] argue for the need to address the root causes of indigenous women’s unjust disadvantage by starting with the training of health professionals, educational inequalities, structural determinants of poverty and designing locally-tailored initiatives. We add to this the need to address the system functions with an equity lens to enable disadvantaged women to receive better care and to contribute to the decolonisation of health systems that damage rather than support indigenous health.

The pay equity gap, unpaid domestic labour and cultural norms of motherhood all also serve to increase gender disparity [49]. Our findings suggest there continue to be barriers to the goal of reproductive justice for all women in New Zealand. Midwives’ stories revealed the difficulties some women experienced when navigating the system, even with support from their midwife. Using the structural violence lens in the midwives’ narratives challenges the neoliberal expectation of individual choice as a marker of success. Situating social disadvantage as an act of an individual failure we can observe the dominant cultural discourse and how it disregards structural factors and fosters circumstances that are likely to worsen, not improve, women’s opportunities when they become pregnant.

### Strengths and Limitations

Using a survey methodology provides both advantages and limitations. Our study resulted in a high response rate with numerous stories from midwives about the different aspects of women’s lives. The anonymity of the survey methodology enabled midwives to provide candid accounts of their experiences of working with women living in socioeconomic deprivation. However, due to the survey methodology, nuance and further exploration of issues was inhibited. In this study, the issue being viewed (women’s socioeconomic deprivation) was through the midwifery perspective, which could be considered subjective. In addition, while we have no ethnicity data from the survey, it is likely that the majority of respondents were of New Zealand European descent (9.8% of the NZ midwifery workforce identifies as Māori and 67.5% as NZ/European) [50], which may have influenced or imposed a Western perspective on the stories told. It is important that other voices are heard. Further work should involve Māori and Pacific researchers, so that the women’s narratives are explored using culturally appropriate epistemologies [34,35]. This would potentially uncover more nuanced understandings of women’s trajectories and needs.

## 5. Conclusions

The structural violence framework has provided a method of identifying the social and institutional structures that expose pregnant women living in social disadvantage to harm. Pregnancy, birth and parenting are foundational to intergenerational health and wellbeing. This study has used midwives’ stories to reveal the deeply troubling everyday realities some women face due to poverty. Despite the stated aim of equitable access to health care for all in policy documents, the current system and social structure continues to perpetuate systemic disadvantage. It is clear that women living in poverty require increased support, funding and resourcing to achieve equitable health outcomes. Ways to do this within maternity, would include increased funding for scans and prescriptions, moving hospital services into the community (including rural regions), providing transport and increasing whanau support. Outside of the maternity system, other urgent issues that need to be addressed, including poverty, housing and food security. Despite the continuity of care maternity model within Aotearoa New Zealand, women living in poverty will continue to face challenges in achieving maternal health equity until the underlying structural and systemic barriers are addressed.

## Figures and Tables

**Table 1:** Overview of themes.

Theme one: Structural disempowerment Reduction in agency—women actively disempowered Psychological disempowerment Reduced agency within family unit Opportunities not conducive to supporting women Lack of access to basic needs
Theme two: Inequitable risk Disadvantaged pregnant women experience exacerbated risks Poverty itself as a medical risk factor Increased stress and mental health concerns Increased risk of family violence and abuse Increased impact of addictions Increased risk through intergenerational transmission Increased risk through stigma and discrimination
Theme three: Neoliberal system Poor housing and access to benefits Rural areas not provided for sufficiently Women in need cannot access acute social services putting them at a higher risk of uplift/family breakdown Women unable to afford basic medical necessities such as scans and medications Focus on the individual/medical rather than the family/holistic health and wellbeing Underfunding of women’s health, unsustainable model Institutionalised racism

## References

[1] Dawson P., Jaye C., Gauld R., Hay-Smith J. (2019). Barriers to equitable maternal health in Aotearoa New Zealand: An integrative review. Int. J. Equity Health.

[2] Kearns R., Moewaka-Barnes H., McCreanor T. (2009). Placing racism in public health: A perspective from Aotearoa/New Zealand. Geojournal.

[3] Ministry of Health (2018). Achieving Equity in Health Outcomes: Highlights of Important National and International Papers.

[4] Ministry of Health Achieving Equity. https://www.health.govt.nz/about-ministry/what-we-do/work-programme-2019-20/achieving-equity.

[5] Sandall J., Soltani H., Gates S., Shennan A., DeVane D. (2016). Midwife-led continuity models versus other models of care for childbearing women. Cochrane Database Syst. Rev..

[6] WHO (2018). World Health Organization WHO Recommendations on Intrapartum Care for a Positive Childbirth Experience.

[7] Ma M.B., Thomson G. (2018). Building capacity and wellbeing in vulnerable/marginalised mothers: A qualitative study. Women Birth.

[8] McLeish J., Redshaw M. (2017). “I didn’t think we’d be dealing with stuff like this”: A qualitative study of volunteer support for very disadvantaged pregnant women and new mothers. Midwifery.

[9] Rayment-Jones H., Murrells T., Sandall J. (2015). An investigation of the relationship between the caseload model of midwifery for socially disadvantaged women and childbirth outcomes using routine data—A retrospective, observational study. Midwifery.

[10] Priday A., McAra-Couper J. (2016). A Successful Midwifery Model for a High Deprivation Community in New Zealand: A Mixed Methods Study. Int. J. Childbirth.

[11] Bartholomew K., Morton S.M.B., Carr P.E.A., Bandara D.K., Grant C.C. (2015). Early engagement with a Lead Maternity Carer: Results fromGrowing Up in New Zealand. Aust. N. Z. J. Obstet. Gynaecol..

[12] Graham R., Masters-Awatere B. (2020). Experiences of Māori of Aotearoa New Zealand’s public health system: A systematic review of two decades of published qualitative research. Aust. N. Z. J. Public Health.

[13] Ratima M., Crengle S. (2013). Antenatal, labour, and delivery care for Māori: Experiences, location within a lifecourse approach, and knowledge gaps. Pimatisiwin A J. Aborig. Indig. Community Heal..

[14] Burgess T.C. (2019). Reconstructing state intervention in pregnancy to empower New Zealand women. Yale J. Law Fem..

[15] Landy C.K., Sword W., Valaitis R. (2008). The Experiences of Socioeconomically Disadvantaged Postpartum Women in the First 4 Weeks at Home. Qual. Health Res..

[16] Ministry of Health (2019). Report on Maternity 2017.

[17] Jakobsen S.P., Overgaard C. (2018). ‘They’ll be judging us’a qualitative study of pregnant women’s experience of being offered participation in a supportive intervention. Midwifery.

[18] Miltenburg A.S., Van Pelt S., Meguid T., Sundby J. (2018). Disrespect and abuse in maternity care: Individual consequences of structural violence. Reprod. Health Matters.

[19] Montesanti S.R., E Thurston W. (2015). Mapping the role of structural and interpersonal violence in the lives of women: Implications for public health interventions and policy. BMC Women’s Health.

[20] Burgess A. (2015). Interconnectedness: The grandparents role in childbearing and parenting. Int. J. Childbirth Educ..

[21] Tantawi-Basra T., Pezaro S. (2020). Supporting childbearing women who are at risk of having their baby removed at birth. Br. J. Midwifery.

[22] Ministry of Health (2007). Maternity Services: Notice Pursuant to Section 88 of the New Zealand Public Health and Disability Act 2000. New Zealand Gazazette.

[23] Homer C.S.E., Davis G.K., Cooke M., Barclay L.M. (2002). Women’s experiences of continuity of midwifery care in a randomised controlled trial in Australia. Midwifery.

[24] Hodnett E.D., Gates S., Hofmeyr G.J., Sakala C. (2017). Continuous support for women during childbirth. Cochrane Database Syst. Rev..

[25] McLachlan H.L., Forster D.A., Davey M.A., Farrell T., Gold L., Biro M.A., Albers L., Flood M., Oats J., Waldenström U. (2012). Effects of continuity of care by a primary midwife (caseload midwifery) on caesarean section rates in women of low obstetric risk: The COSMOS randomised controlled trial. BJOG Int. J. Obstet. Gynaecol..

[26] Winter Y. (2012). Violence and Visibility. New Political Sci..

[27] Galtung J. (1969). Violence, Peace, and Peace Research. J. Peace Res..

[28] De Maio F., Ansell D. (2018). “As Natural as the Air Around Us”: On the Origin and Development of the Concept of Structural Violence in Health Research. Int. J. Health Serv..

[29] Smith L.T. (2012). Decolonizing Methodologies: Research and Indigenous Peoples.

[30] Smith N. (2019). ‘Carried off in their hundreds’: Epidemic diseases as structural violence among Indigenous peoples in Northwestern Australia. Hist. Anthr..

[31] Gamlin J., Holmes S. (2018). Preventable perinatal deaths in indigenous Wixárika communities: An ethnographic study of pregnancy, childbirth and structural violence. BMC Pregnancy Childbirth.

[32] Sánchez-Sauco M.F., Villalona S., Ortega-García J.A. (2019). Sociocultural aspects of drug dependency during early pregnancy and considerations for screening: Case studies of social networks and structural violence. Midwifery.

[33] Braun V., Clarke V., Boulton E., Davey L., McEvoy C. (2020). The online survey as a qualitative research tool. Int. J. Soc. Res. Methodol..

[34] Milaney K., Lockerbie S.L., Fang X.Y., Ramage K. (2019). The role of structural violence in family homelessness. Can. J. Public Health.

[35] Came H., McCreanor T., Simpson T. (2016). Health activism against barriers to indigenous health in Aotearoa New Zealand. Crit. Public Health.

[36] Ratima M. (2010). Māori Health Promotion a Comprehensive Definition and Strategic Considerations.

[37] Onuzo U., Garcia A.F., Hernandez A., Peng Y., Lecoq T. (2013). Intergenerational Equity Understanding the Linkages between Parents and Children: A Systematic Review.

[38] Allison D., Budhwani H. (2014). Faculty Opinions recommendation of the intergenerational transmission of inequality: Maternal disadvantage and health at birth. Fac. Opin. Publ. Peer Rev. Biomed. Lit..

[39] Talmon A., Horovitz M., Shabat N., Haramati O.S., Ginzburg K. (2019). “Neglected moms”—The implications of emotional neglect in childhood for the transition to motherhood. Child Abus. Negl..

[40] Buss C., Entringer S., Moog N.K., Toepfer P., Fair D.A., Simhan H.N., Heim C.M., Wadhwa P.D. (2017). Intergenerational Transmission of Maternal Childhood Maltreatment Exposure: Implications for Fetal Brain Development. J. Am. Acad. Child Adolesc. Psychiatry.

[41] Lee J., Seon J. (2019). Intergenerational transmission of maternal poverty to self-esteem among young adult children: The role of employment. Child. Youth Serv. Rev..

[42] Strulik H. (2019). Myopic misery: Maternal depression, child investments, and the neurobiological poverty trap. Macroecon. Dyn..

[43] McRae D.N., Janssen P.A., Vedam S., Mayhew M., Mpofu D., Teucher U., Muhajarine N. (2018). Reduced prevalence of small-for-gestational-age and preterm birth for women of low socioeconomic position: A population-based cohort study comparing antenatal midwifery and physician models of care. BMJ Open.

[44] Vedam S., Stoll K., MacDorman M., Declercq E., Cramer R., Cheyney M., Fisher T., Butt E., Yang Y.T., Kennedy H.P. (2018). Mapping integration of midwives across the United States: Impact on access, equity, and outcomes. PLoS ONE.

[45] PMMRC (2018). Twelfth Annual Report of the Perinatal and Maternal Mortality Review Committee: Reporting Mortality 2016.

[46] Moewaka Barnes H., Moewaka Barnes A., Baxter J., Crengle S., Pihama L., Ratima M., Robson B. (2013). Hapū Ora: Wellbeing in the Early Stages of Life.

[47] Chopel A.M. (2013). Reproductive health in indigenous Chihuahua:giving birth ‘alone like the goat’. Ethn. Health.

[48] Gamlin J.B., Hawkes S.J. (2014). Pregnancy and birth in an indigenous Huichol community: From structural violence to structural policy responses. Cult. Health Sex..

[49] Peters S., Woodward M., Jha V., Kennedy S., Norton R. (2016). Women’s health: A new global agenda. BMJ Glob. Health.

[50] Midwifery Council (2019). Midwifery Workforce Survey.

